# Mechanical Properties and Microstructure of Hot-Pressed Silica Matrix Composites

**DOI:** 10.3390/ma15103666

**Published:** 2022-05-20

**Authors:** Weili Wang, Jianqi Chen, Xiaoning Sun, Guoxun Sun, Yanjie Liang, Jianqiang Bi

**Affiliations:** 1 Key Laboratory for Liquid-Solid Structural Evolution and Processing of Materials, Ministry of Education, School of Materials Science & Engineering, Shandong University, Jinan 250061, China; cjcq1332511377@163.com (J.C.); sava1982@163.com (X.S.); sunguoxun0228@163.com (G.S.); yanjie.liang@sdu.edu.cn (Y.L.); bjq1969@163.com (J.B.); 2 Suzhou Institute of Shandong University, Suzhou 215123, China

**Keywords:** silica, boron nitride, alumina platelets, mechanical property, microstructure

## Abstract

Silica is one of the most widely used ceramics due to its excellent chemical stability and dielectric property. However, its destructive brittle nature inhabits it from wider application as a functional ceramic. An improvement in toughness is a challenging topic for silica ceramic, as well as other ceramics. In the paper, silica ceramic with different types of boron nitride powders and alumina platelets was fabricated by hot-pressing. Introduction of the additives had great influence on the composites’ mechanical properties and microstructure. The silica matrix composite containing micro-sized boron nitride powders possessed the best mechanical properties, including the bending strength (134.5 MPa) and the fracture toughness (1.85 Mpa·m^1/2^). Meanwhile, the introduction of alumina platelets combined with boron nitride nanosheets achieved an effective enhancement of fracture toughness while maintaining the bending strength. Compared with the monolithic silica, the composite with simultaneous addition of alumina platelets and boron nitride nanosheets had a fracture toughness of 2.23 Mpa·m^1/2^, increased by approximately 27% (1.75 Mpa·m^1/2^). The crack deflection and platelet pullout were contributing to enhancement of the fracture toughness. The improved mechanical properties, combined with the intrinsic excellent dielectric and chemical properties, make the silica matrix composites promising wave transparent and thermal protection materials.

## 1. Introduction

Silica (SiO_2_) is an important structural and functional ceramic for many applications because of its excellent properties, such as good resistance to corrosion and thermal shock, low dielectric permittivity versus temperature, and low thermal conductivity, etc. [1,2,3,4,5]. However, the poor mechanical properties, especially low fracture toughness, restrict its applications since the reliability is the first concerned factor in engineering fields. In an effort to solve this problem, various kinds of materials as secondary phases have been employed into the SiO_2_ matrix. These additives are expected to improve the mechanical properties of SiO_2_ ceramic without deterioration of its functional properties. Up to now, fiber, silicon nitride (Si_3_N_4_), and zirconia (ZrO_2_) have attempted to reinforce SiO_2_, especially fused SiO_2_ [6,7,8,9,10,11]. For example, Wan et al. prepared ZrO_2_-reinforced fused SiO_2_ using a low-toxic gelcasting method followed by pressureless sintering [6]. The experimental results showed that the addition of ZrO_2_ improved the composites’ mechanical strength significantly. In another research report, the addition of Si_3_N_4_ enhanced both ambient strength and fracture toughness of fused SiO_2_ [7].

With the development of nanomaterials, carbon nanotubes (CNTs) and graphene have also been employed as reinforcements to improve SiO_2_ ceramic’s mechanical properties. Reduced graphene oxide/fused SiO_2_ composites were fabricated by spark plasma sintering (SPS), and the increased conductivity and Vickers hardness were achieved [11]. Arvanitelis et al. prepared CNT/SiO_2_ composites by a colloidal processing and pressureless sintering, and homogenous dispersion of CNTs in the matrix and high density of the sintered bodies were obtained [12].

Among the additives for SiO_2_ ceramic, hexagonal boron nitride (*h*-BN) is an effective reinforcement due to some similar properties to SiO_2_, such as low dielectric dissipation fraction and high dielectric strength [13]. It is reported that the BN/SiO_2_ composites exhibited higher strength, fracture toughness, and other functional properties than pure SiO_2_ [14,15,16]. Additionally, *h*-BN powders are generally a plate-like shape, which is conducive to utilizing the improvement effect. The BN-reinforced SiO_2_ composites are regarded as very promising candidate materials as wave transparent thermal protection parts [16].

Besides BN particles, BN nanotubes (BNNTs) and BN nanosheets (BNNSs) have also been introduced into SiO2 matrix, and the influence of the nanomaterials has been investigated. BNNTs have more advantages than CNTs, including good chemical stability, high oxidation temperature, and low dielectric constant, which make them more suitable as ceramic reinforcements [17,18,19]. Many attempts have been done to strengthen ceramics, using BNNTs as a secondary phase. For example, the experimental results of Huang et al. showed that the addition of BNNTs dramatically enhanced the high-temperature superplastic deformation of engineering ceramics [20]. Li et al. fabricated BNNTs/Si_3_N_4_ composites by hot-pressing method, and the fracture toughness of Si_3_N_4_ was enhanced significantly [21]. Concerning the studies on BNNTs/SiO_2_ composites, it is reported that the BNNTs reinforced SiO_2_ composites exhibited excellent mechanical properties and low dielectric constant [22]. BNNSs are one of the most attractive 2D nanomaterials, not only due to the structural similarity to graphene, but also owing to their unique properties [23,24,25]. The advantages of BNNSs make them promising candidate materials to reinforce ceramics, applying in more severe environment than carbon nanomaterials. Lee et al. proposed BN nanoplatelets as the toughening reinforcement components to Si_3_N_4_ ceramic [26]. The fracture toughness, bending strength, and tribological properties of the BNNP/Si_3_N_4_ nanocomposite were increased up to a point. When BNNSs were employed as reinforcement for fabrication of fused SiO_2_ matrix composites, both bending strength and fracture toughness were improved [27].

Apart from the additives listed above, Du et al. fabricated SiO_2_ composites containing BN powders (BN nanoparticles and BNNTs) and alumina (Al_2_O_3_) [13]. The research findings were very interesting, concluding that a significant enhancement of mechanical properties was obtained. The coupling effect of the combined additives is expected to improve the mechanical properties and the functional performance of SiO_2_. However, up to date, there are still few literatures about SiO_2_ matrix composites with combined secondary phases. Therefore, more attempts are encouraged, and the experimental results will be promising.

In this study, SiO_2_ matrix composites were fabricated by hot-pressing, in which three types of BN powders (micro-sized BN, nano-sized BN, and BNNSs) and Al_2_O_3_ platelets were introduced as the secondary phases. Both the BN powders and Al_2_O_3_ platelets influenced the microstructure and mechanical properties, including bending strength and fracture toughness, of the hot-pressed SiO_2_ matrix composites. Based on the microstructure observation, the relationship between composition, microstructure, and mechanical property was established, and the strengthening and toughening mechanisms were discussed.

## 2. Materials and Methods

The SiO_2_ powders purchased from Sinopharm Chemical Reagent Co., Ltd., Shanghai, China, were used as the raw materials without further purification. Three types of BN powders, including micro-sized BN, nano-sized BN, and BNNSs powders, were employed as additives of SiO_2_ ceramic in this work. The micro-sized BN (BN_m_) and nano-sized BN (BN_n_) powders were provided by Shanghai Macklin Biochemical Co. Ltd., Shanghai, China, with a purity of 99.9%. BNNSs were bought from Nanjing XFNANO Materials Tech Co., Ltd., Nanjing, China, with a purity of 98 wt.% claimed by the supplier. In addition, α-Al_2_O_3_ platelets (Ronaflair white sapphire, Merck, Darmstadt, Germany) were also used to reinforce the SiO_2_ ceramic. Four composite samples were fabricated, which were designated as S2 (SiO_2_ + 1.0 wt.% BNNSs), S3 (SiO_2_ + 1.0 wt.% BN_m_), S4 (SiO_2_ + 1.0 wt.% BN_n_), and S5 (SiO_2_ + 1.0 wt.% BNNSs + 10.0 wt.% α-Al_2_O_3_ platelets). Additionally, a pure SiO_2_ ceramic, named as S1, was also fabricated as a reference. The number and the exact composition of samples are shown in Table 1.

Typically, the composite powders were weighed, and ball milled for 8 h with ethanol as the ball-milling medium. Before mixing by ball-milling, the three types of BN powders were dispersed in 150 mL ethanol by sonication for 2 h. Next, the ball milled slurry was poured into a container and placed in an oven at 80 °C to remove the ethanol completely. After that, the powders were collected, and put in a graphite die with a diameter of 42 mm. The hot-pressing was conducted in a High-Multi 5000 furnace (Fuji Dempa Kogyo Co., Ltd., Osaka, Japan) at 1450 °C for 1 h under a pressure of 30 Mpa in an argon atmosphere.

**Figure 1:**
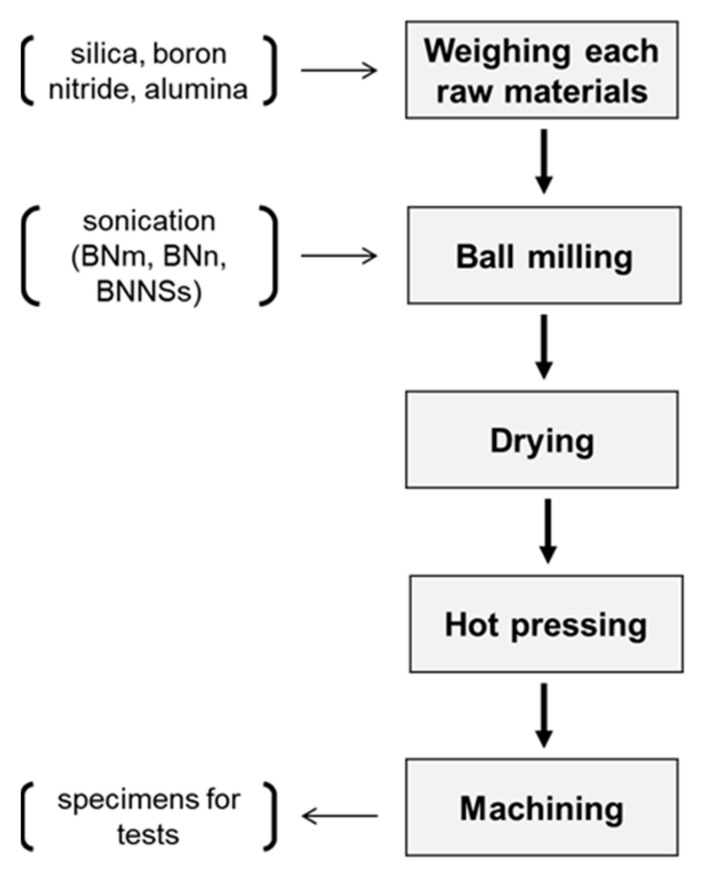
Flowchart of the sample preparation.

The hot-pressed samples were polished for the relative density measurement via the Archimedes method (ASTM B962-17 standard), in which distilled water was used as the medium. The density of 2.30 g/cm^3^, 2.27 g/cm^3^, and 3.97 g/cm^3^ was adopted for SiO_2_, BN, and Al_2_O_3_ as the theoretical densities for density calculation. Then, the samples were cut into test bars accurately. Before mechanical property test, the bars were polished by B_4_C powders to remove the possible damage produced in the cut process. The bending strength and fracture toughness tests were conducted on a CMT6203 universal testing machine (MTS Systems (China) Co., Ltd., Shenzhen, China). The bar with the dimension of 3.0 mm (width) × 4.0 mm (thickness) × 30.0 mm (length) were used for bending strength test by the three-point bending method (ASTM C1161-18 standard). The 2.0 mm (width) × 4.0 mm (thickness) × 30.0 mm (length) bars were prepared for the fracture toughness test using single-edge notched beam (SENB) method (ASTM C1421-01b standard). All the bars had a notch of 0.3 mm (width) × 2.0 mm (depth) in size in the center. During the bending strength test, the load speed was 0.5 mm/s. By contrast, the load speed was 0.05 mm/s for the fracture toughness measurement. The span length of 20.0 mm was determined for both bending strength and fracture toughness tests. The bending strength and fracture toughness values are calculated by Equation (1) and Equations (2) and (3).

(1) $$\sigma_{f}=\frac{3PL}{2bW^{2}}$$

where *σ_f_* is the bending strength of the samples, *P* is the load at the fracture point, *L* is the span length, *b* is the width of the sample, *W* is the thickness of the sample.

(2) $$K_{IC}=Y\frac{3PL}{2bW^{2}}\sqrt{a}$$

(3) $$Y=1.93-3.07\times \left(\frac{a}{W}\right)+14.53\times {\left(\frac{a}{W}\right)}^{2}-25.11\times {\left(\frac{a}{W}\right)}^{3}+25.8\times {\left(\frac{a}{W}\right)}^{4}$$

where *K_IC_* is the fracture toughness of the sample, *a* is the notch depth of the sample, *b* and *W* are the width and thickness of the sample, respectively. Generally, the average values of four specimens were calculated as the final measured values of bending strength and fracture toughness. The processing steps of the silica matrix composites are outlined in Figure 1.

The phases of the composites were determined by a D/max-RA X-ray diffractometer (XRD) (Rigaku, Tokyo, Japan) with Cu Kα X-ray source. Meanwhile, the microstructure evolution of the composites’ fracture surfaces was observed by a Hitachi SU-70 type scanning electron microscope (SEM) (Hitachi, Tokyo, Japan) with an energy dispersive spectrometer (EDS) attached. An optical microscope (LW600LT, Shanghai Cewei Optoelectronic Technology Co., Ltd., Shanghai, China) was employed for the crack path observation in this work.

## 3. Results and Discussion

**Figure 2:**
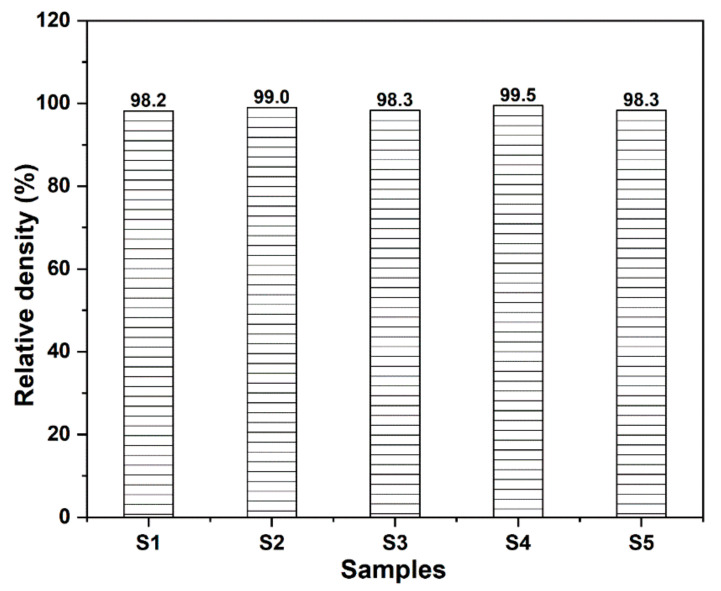
Relative density of the samples.

The influence of the additives on the properties of the SiO_2_ matrix composites were investigated. The variation tendency of relative density is shown in Figure 2. In the measurement process, the polished samples with a diameter of 42 mm and a thickness of 4 mm are used for weighing. It can be seen from Figure 2 that all the samples have a relatively high volume density (>98%). Generally, the addition of nanomaterials as reinforcements may result in decrease of relative density. However, the addition of additives has a slight influence on the relative density of samples in this work because of the small number of additives in the SiO_2_ matrix. At the end of sintering, some residual pores cannot be removed easily, which will become the closed pores left inside the sintered bodies. Due to a very small amount of BN dispersed in the matrix, refined grains will be obtained, leading to removal of some residual pores at the same time. Therefore, all the samples are well-densified, and S2, S3, S4, and S5 possess a slight increase of relative density in comparison with S1.

**Figure 3:**
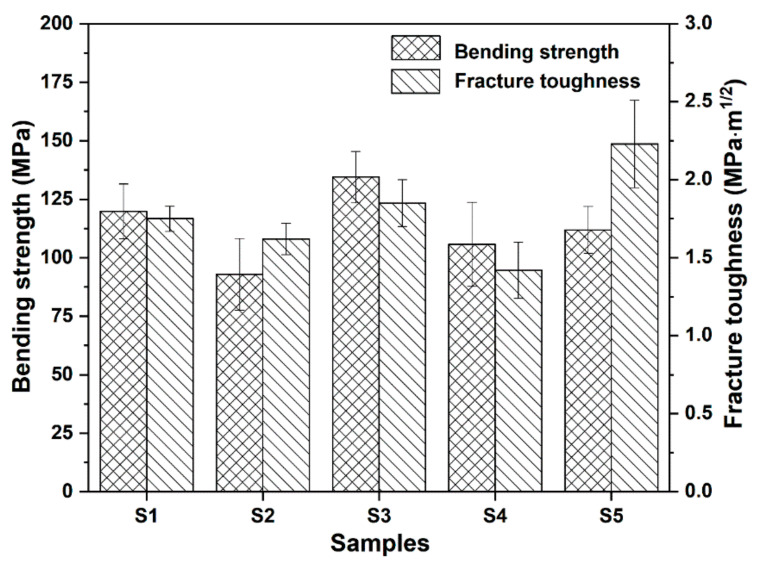
Mechanical properties of the samples.

Figure 3 shows the bending strength and fracture toughness of the SiO_2_ matrix composites with different additives. The sample bars with a definite dimension (see Materials and Methods) are used in the tests, and the bending strength and fracture toughness are calculated according to Equations (1)–(3). It can be seen in Figure 3 that the introduction of BN powders and Al_2_O_3_ platelets significantly affects the mechanical properties. Among the samples using BN powders as additives, S3 possesses the best mechanical properties compared with pure SiO_2_ ceramic (S1), S3, which contains 1.0 wt.% BN_m_, and exhibits a slight increase in bending strength (134.5 Mpa vs. 119.8 Mpa) and fracture toughness (1.85 Mpa·m^1/2^ vs. 1.75 Mpa·m^1/2^). Other BN powders, especially BNNSs, are introduced into the matrix, which reduces the mechanical properties unexpectedly. In comparison with S4, S2 has higher fracture toughness, while S4 possesses better bending strength. Notably, when BNNSs and Al_2_O_3_ platelets are incorporated simultaneously, the fracture toughness of the composite (S5) increases, from 1.75 Mpa·m^1/2^ to 2.23 Mpa·m^1/2^, while there is a slight decrease in bending strength (119.8 Mpa vs. 111.9 Mpa). For ceramics, their bending strength mainly depends on balance of grain size and defect, while their fracture toughness mainly depends on crack propagation resistance. The addition of BN powders causes grain refinement, which is good for increase of bending strength. However, agglomerates of BNNSs and BN_n_ will emerge easily owing to their small particle size, leading to decrease of bending strength. Therefore, reduction in bending strength of S2 and S4 is the balanced results of grain size and BN agglomerates. By contrast, addition of Al_2_O_3_ platelets us improve the crack propagation resistance significantly in S5, so its fracture toughness is obviously enhanced. The microstructure analysis in the next section will provide exact evidence for the variation of mechanical properties.

**Figure 4:**
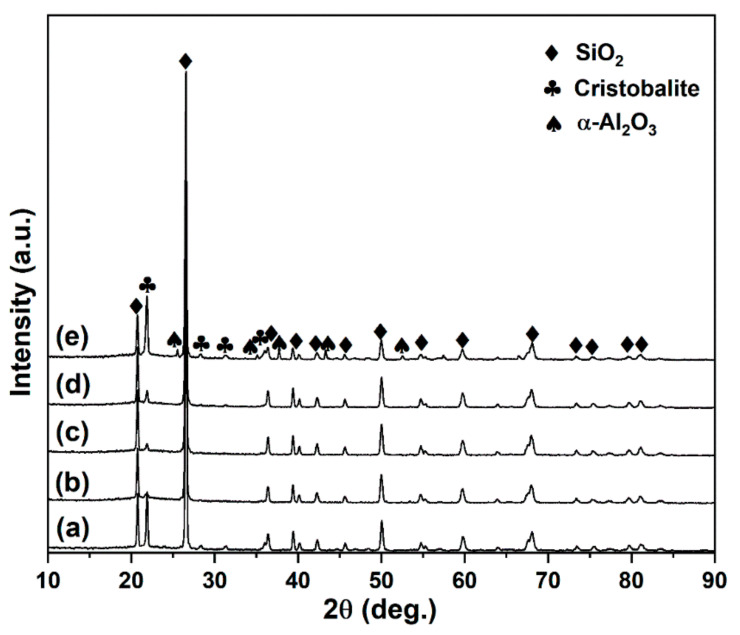
XRD patterns of the samples, (**a**) S1; (**b**) S2; (**c**) S3; (**d**) S4; (**e**) S5.

The effective phase and microstructure analysis can provide more information about the mechanical property variation. Figure 4 illustrates the XRD patterns of the hot-pressed SiO_2_ matrix composites. For all samples, in addition to the main phase quartz (SiO_2_, JCPDS 86-1560), cristobalite phase (JCPDS 76-0936) can also be detected. It is reported that the critical transformation temperature of quartz to cristobalite is 1300 °C~1350 °C [28]. The existence of cristobalite is normal since all the samples were sintered at 1450 °C in this work. However, the formation of cristobalite may induce microcracks, causing deterioration of the mechanical properties [29]. The intensity of the cristobalite characteristic peaks decreases obviously when three types of the BN powders are introduced into the matrix (Figure 4b–d). It is indicated that the addition of BN powders can restrain the transformation of quartz to cristobalite. The suppression effect of quartz→cristobalite transformation may be helpful to avoid severe degradation of mechanical properties. When BNNSs and Al_2_O_3_ platelets are added together, the characteristic peaks of corundum (α-Al_2_O_3_, JCPDS76-0144) can be detected, except for those of quartz and cristobalite (Figure 4e). Moreover, the simultaneous addition of BNNSs and Al_2_O_3_ platelets cannot effectively inhibit the quartz→cristobalite transformation. Owing to the low content of BN powders (1.0 wt.%) in the samples, the characteristic peaks of BN are not observed in the XRD patterns.

**Figure 5:**
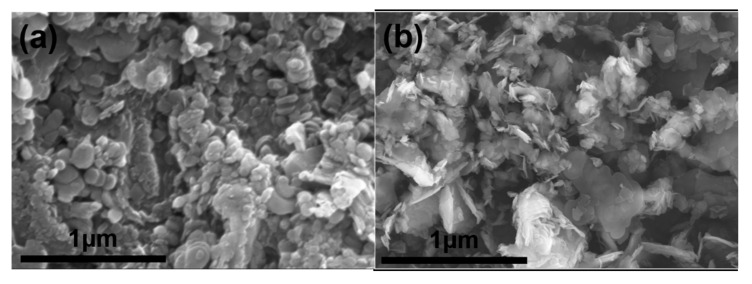

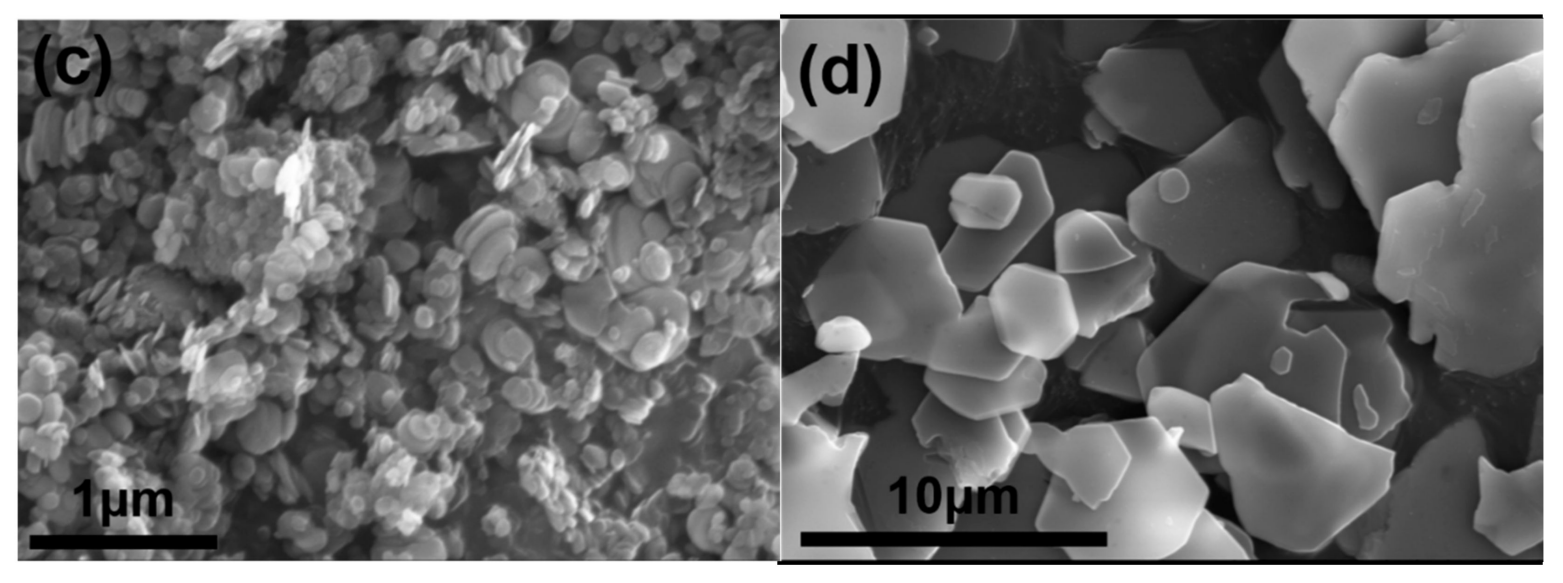
SEM images of (**a**) BNNSs; (**b**) BN_m_; (**c**) BN_n_; (**d**) Al_2_O_3_ platelet.

Structural features of all the additives can be found in the typical SEM images in Figure 5. From Figure 5a,c, it is can be seen that BNNSs and BN_n_ have very small particle size, <200 nm for BNNSs and 200–500 nm for BN_n_, respectively. Achieving good dispersibility of the added nanomaterials in matrix is very tough. Therefore, the decrease of the mechanical properties of S2 and S4 may be related to the particle agglomeration. A wide particle size range of BN_m_ powders, from ~1 μm to several μm, can be observed in Figure 5b. In Figure 5d, the platelet-like Al_2_O_3_ with unregular morphology can be seen clearly.

**Figure 6:**
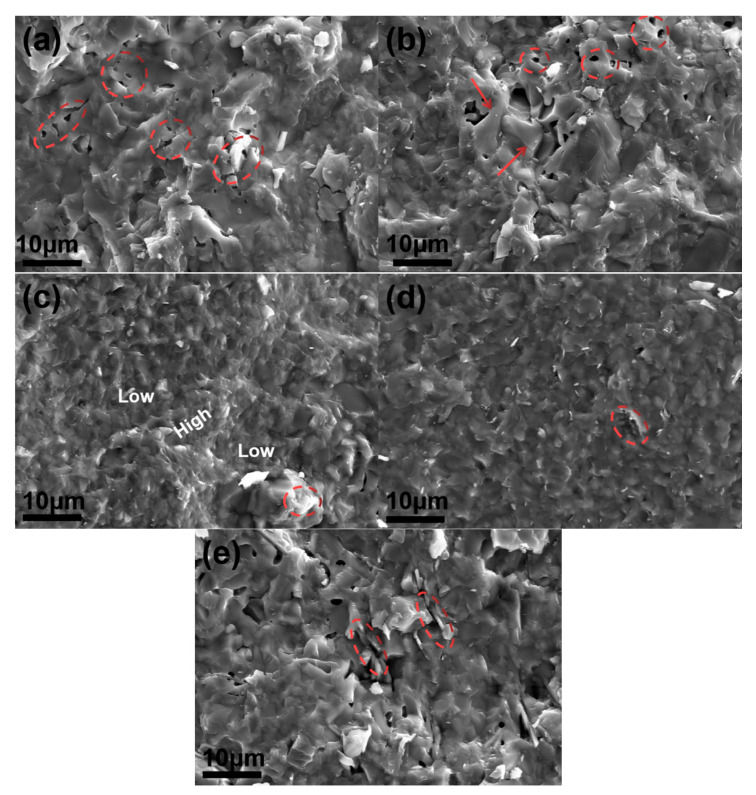
SEM images of fracture surface, (**a**) S1; (**b**) S2; (**c**) S3; (**d**) S4; (**e**) S5.

The addition of BN powders and Al_2_O_3_ platelets (Figure 5) affects the microstructure, and has a great influence on the mechanical properties of the SiO_2_ matrix composites (Figure 3). The coupling effect of BN powders and Al_2_O_3_ platelets is conducive to the improvement of mechanical properties, which has proved to be useful additives in the SiO_2_ matrix composites. The typical SEM images, showing the features of the fracture surface of the composites, are displayed in Figure 6. Figure 6a shows a wave-like fracture surface of the monolith SiO_2_, in which some closed pores are embedded as the circle marked. Therefore, the relative density of S1 is 98.2% displayed in Figure 2, because of the closed pores inside the densified body. Nevertheless, the morphology is changed with the introduction of additives. The decrease of grain size and some residual large grains can be observed in Figure 6b, when BNNSs are introduced (S2). Although the number of pores decreased, some closed pores can be still observed on the surface (red circles). Besides, some abnormal large grains can also be seen as the arrow marked in Figure 6b. The large grains and the pores around them form a defect in S2, which contributes to reduction in bending strength. With the addition of BN_m_ and BN_n_ powders, the grain size also decreases in Figure 6c,d compared with the monolith (Figure 6a). Notably, the fracture surface of S3 is more compact, leaving no obvious closed pores and less BN_m_ dots, as seen in Figure 6c. In addition, S3 features with fluctuant fracture surface, as indexed in Figure 6c, means the existence of crack propagation during fracturing. Meanwhile, the torn grain can also be seen in the fracture surface (the circle in the figure). The fluctuant fracture surface and torn grain are beneficial to energy dissipation, leading to enhancement of mechanical properties for S3. It is known that the addition of reinforcement phase, especially nanomaterials, can restrain grain growth more or less during sintering. Therefore, the viscous flow of SiO_2_ and grain growth will be inhibited by BN powders, leading to relatively smaller particle size of S2, S3, and S4. On the one hand, the addition of BNNSs and BN_n_ may refine grain size because of their nanometer size; on the other hand, their hard dispersibility may result in the agglomeration in the matrix, and the decrease of mechanical property. Therefore, compared with S2 and S4, S3, which contains 1.0 wt.% BN_m_, possesses the proper grain size and less reinforcement phase agglomeration, achieving increased bending strength and fracture toughness. In addition to BN_n_ agglomeration as marked in Figure 6d, the fracture surface of S4 seems flat, indicating relatively weak crack propagation resistance during fracturing. Therefore, both bending strength and fracture toughness for S4 decrease unsurprisingly. When BNNSs and Al_2_O_3_ platelets are used in S5, its fractured feature is similar to that of S2, and Al_2_O_3_ platelets can be easily found on the fracture surface as well as some closed pores, as shown in Figure 6e. Although the bending strength of S5 decreased slightly, the pull-out of Al_2_O_3_ platelets (the residual holes marked by the circles) and the crack propagation resistance originating from blocking of the platelets contribute to the obvious improvement of S5′s fracture toughness.

**Figure 7:**
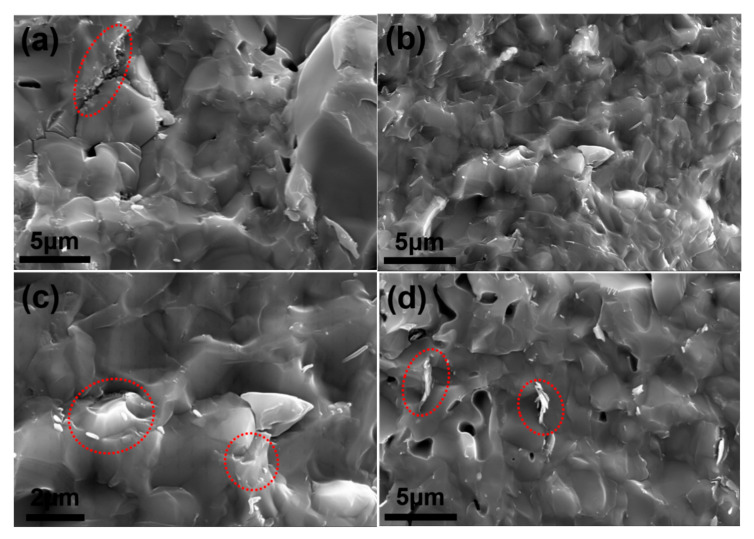
SEM images of fracture surface, (**a**) S2; (**b**,**c**) S3; (**d**) S4.

Figure 7 shows the SEM images with higher magnification of the composites with BN powders, which can provide more information about fracture behavior and mechanical property variation. The agglomeration of BNNSs can be found on the fracture surface as the circle marked in Figure 7a. The agglomeration forms a loose defect, which acts as a pore in the matrix, leading to deterioration of the mechanical properties especially the bending strength. The Hall–Petch relationship (Equation (4)) is a classic equation in metals, which shows us that finer grain size is beneficial to production of higher bending strength. A similar relationship between grain size and bending strength is proved to exist in ceramics, that is, the finer grain size is beneficial to higher bending strength [30].

(4) σb=σ0+κ·d−12

where *σ_b_* is the strength of sample, *σ*_0_ and *κ* are material constants and independent of grain size, and *d* is grain size. Finally, the bending strength is an equilibrium result of the defect and grain size effects in ceramics. Obviously, the influence of defects in S2, including pores and BNNSs agglomeration, is stronger than that of refined grain size, leading to a low mechanical property. By contrast, S3 shows an increased mechanical property, both bending strength and fracture toughness. Due to the larger particle size, the number of BN_m_ particles in the composite is much less than those of BNNSs and BN_n_ under the same mass percentage. Therefore, agglomeration of BN_m_ is rarely observed on the surface in Figure 7b. Moreover, there are no obvious closed pores. The combined effect of refined grain size and less defects increases the bending strength and fracture toughness of S3. As shown in Figure 7c, a very short length of BN_m_ exists on the grain boundaries, indicating strong interfacial bonding between the matrix and the reinforcement. Torn grains around the short BN_m_ signify that the fracture mode is trans-granular fracture rather than inter-granular fracture. Overall, the addition of BN_m_ into the matrix is conducive to refine the grain size and strengthen the grain boundary, which will consume more energy during the fracture process. Concerning S4, which contains BN_n_ as reinforcement phase, it also possesses reduced mechanical properties compared with the monolithic SiO_2_. From Figure 7d, the defects originating from closed pores and BN_n_ agglomeration (as the circle marked) can be easily found on the surface, which results in a negative effect on the mechanical properties.

**Figure 8:**
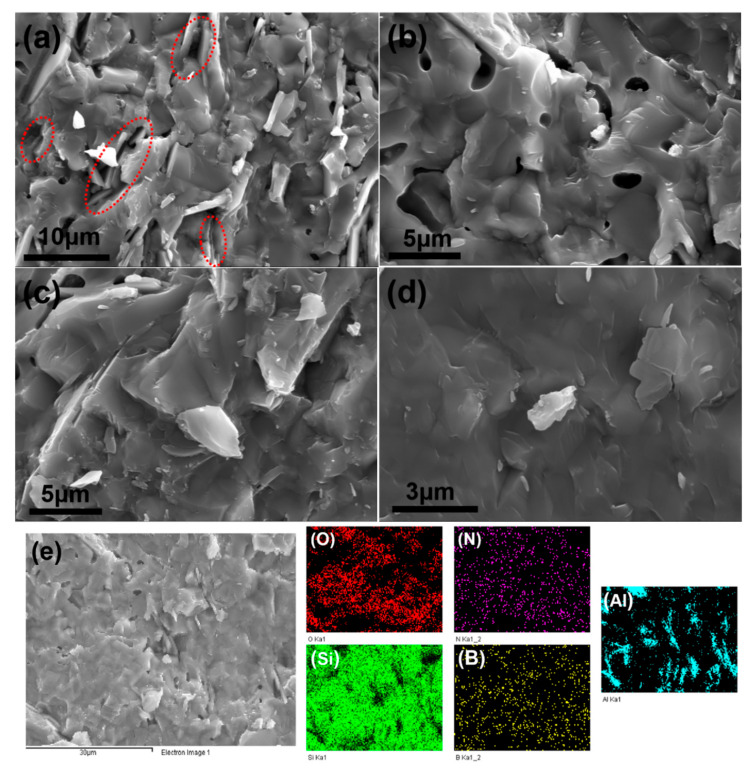
(**a**–**d**) SEM images and (**e**) EDS mapping of the fracture surface of S5.

**Figure 9:**
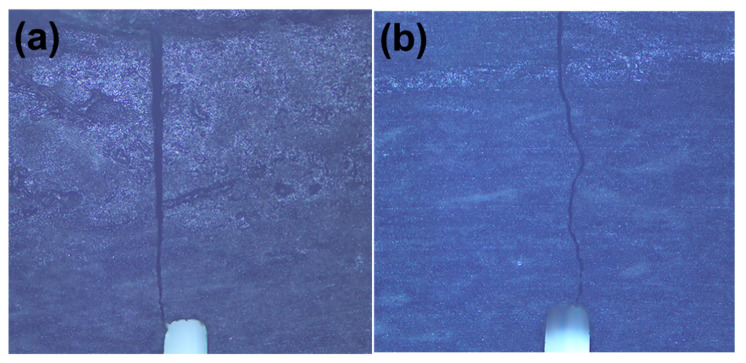
Optical images of crack path, (**a**) S1; (**b**) S5.

When both BNNSs and Al_2_O_3_ platelets are added into the matrix (S5), the bending strength remains nearly constant in comparison with the pure SiO_2_, while a gratifying fracture toughness is achieved as discussed in Figure 3. The SEM images in Figure 8 give more information to investigate the mechanism. First, the fracture surface is very rough as shown in Figure 8a, which means that the cracks experience a tortuous propagation during fracture. Second, the Al_2_O_3_ platelets pull-out can also be found on the surface. The holes (as marked in Figure 8a) originating from pull-out of the Al_2_O_3_ platelets are visible, which have a different shape from closed pores. Third, although the addition BNNSs and Al_2_O_3_ platelets cannot remove the closed pores in S5 completely (Figure 8b), the effective bonding between BNNSs and SiO_2_ matrix causes intra-crystalline rupture, as revealed in Figure 8c. The trans-granular fracture mode helps to dissipate fracture energy compared with inter-granular fracture. Moreover, from the SEM image with a higher magnification in Figure 8d, it can be seen that BNNSs mainly locate the grain boundaries, which can affect abnormal grain growth. All the influencing factors mentioned above, including tortuous crack path, pull-out of Al_2_O_3_ platelets, intra-crystalline rupture, and grain refinement owing to BNNSs addition, work together to contribute the enhancement of the fracture toughness for S5. The EDS analysis is displayed in Figure 8e, and the element mapping of B and N indicates that BNNSs distribute relatively uniformly in the whole matrix. The element mapping of Al shows that Al_2_O_3_ platelets are not distributed homogeneously, however, they individually distribute in the matrix abnormally. The abnormal distribution inhibits and deflects the crack propagation path effectively. As mentioned above, the toughening mechanisms originating from BNNSs and Al_2_O_3_ platelets contribute the enhancement of fracture toughness of S5. Therefore, the samples exhibit a different crack propagation mode during fracture toughness test. The crack paths of S1 and S5 can be seen in Figure 9. Due to the intrinsic brittleness of SiO_2_ ceramic, S1 shows a very straight crack propagation path, as shown in Figure 9a. Comparatively, S5 displays a tortuous propagation path clearly in Figure 9b. Consequently, the coupling effect of BNNSs and Al_2_O_3_ platelets gives rise to an increased fracture toughness.

## 4. Conclusions

In summary, SiO_2_ matrix composites with different additives, including BN_m_ and BN_n_ powders and BNNSs sand Al_2_O_3_ platelet, were fabricated by hot-pressing. The addition of the additives greatly influenced the microstructure and mechanical properties. The introduction of BN additives suppressed the phase transformation from quartz to cristobalite, avoiding severe deterioration of mechanical property. Particularly, the incorporation of BN_m_ powders was beneficial to remove closed pores in the sintered body, leading to a slight increase of both bending strength (134.5 MPa vs. 119.8 MPa) and fracture toughness (1.85 MPa·m^1/2^ vs. 1.75 MPa·m^1/2^). The addition of BN_n_ powders and BNNSs resulted in a decrease of mechanical properties because of pore defects and agglomeration of BN_n_ powders and BNNSs. When the Al_2_O_3_ platelets and BNNSs were incorporated simultaneously, the SiO_2_ composite exhibited an increase of fracture toughness, from 1.75 MPa·m^1/2^ to 2.23 MPa·m^1/2^. Al_2_O_3_ platelets played a vital role on the fracture toughness improvement, principally because of crack deflection and platelet pullout. Therefore, it is believed that the SiO_2_ matrix composites are a promising candidate to be used as wave transparent and thermal protection material because of their inherent low dielectric constant and good chemical stability as well as their enhanced mechanical properties. The composites are useful materials for developing high-performance ceramic parts, such as wave transparent windows and nose tips of aircraft.

## Figures and Tables

**Table 1:** Composition of the samples.

No.	SiO_2_ (wt.%)	BNNSs (wt.%)	BN_m_ (wt.%)	BN_n_ (wt.%)	Al_2_O_3_ Platelet (wt.%)
S1	100	-	-	-	-
S2	99.0	1.0	-	-	-
S3	99.0	-	1.0	-	-
S4	99.0	-	-	1.0	-
S5	89.0	1.0	-	-	10.0

## Data Availability

All the supporting and actual data are presented in the manuscript.
